# Predicting clinical outcomes in chronic hepatitis B patients receiving nucleoside analogues and pegylated interferon alpha: a hematochemical and clinical analysis

**DOI:** 10.1186/s12879-024-10057-0

**Published:** 2024-10-13

**Authors:** Shuang Xu, Xiao-Ting Ye, Dong Zhang, Pu Dong, Yang-He Wu, Chen-Wei Pan

**Affiliations:** 1 Department of infectious Diseases, The Third Affiliated Hospital of Wenzhou Medical University, Wenzhou, China; 2 Department of infectious Diseases, The Second Affiliated Hospital and Yuying Children’s Hospital, of Wenzhou Medical University, Wenzhou, China

**Keywords:** Chronic Hepatitis B, HBsAg clearance, Pegylated interferon alpha, Nucleos(t)ide analogs, Predictive model

## Abstract

**Background:**

The best antiviral treatment for chronic hepatitis B (CHB) poses a complex challenge. The treatment effect of the combination of nucleoside analogues (NAs) and pegylated interferon alpha (PegIFN) was still in debate.

**Methods:**

We studied patients treated with NAs and PegIFN-2b at our institution from November 2019 to January 2022. Logistic regression identified independent factors influencing clinical cure. The predictive accuracy of the formula was assessed using the Receiver operating characteristic (ROC) curve at different time points (before therapy, 12 weeks, and 24 weeks into treatment).

**Results:**

A total of 120 patients were enrolled in the final analysis. Among the cohort of patients under study, 71 (59.1%) patients had clinical cure while 49 (40.9%) patients did not. Hepatitis B surface antigen (HBsAg) at baseline and age were the powerful variables predicting the clearance of HBsAg. The area under the ROC (AUC) was 0.907 for pre-treatment predictive model, 0.958 for 12-week predictive model and 0.747 for 24-week predictive model.

**Conclusion:**

This study provided predictive formulas for clinical cure, offering valuable insights for CHB treatment. PegIFN and NAs exhibited efficacy. Future research that explores additional factors, such as HBV genotype, in a larger cohort study is needed.

## Introduction

Chronic hepatitis B (CHB) is a prevalent viral liver disease, leading to liver cirrhosis and liver cancer, affecting over 300 million people globally with a prevalence of 4.1% in 2019 [1, 2]. In China alone, approximately 70 million individuals are chronically infected with HBV, with 20 million diagnosed with CHB [3]. The persistence of covalently closed circular DNA (cccDNA) within hepatocytes is a crucial aspect of CHB infection [4]. Hepatitis B surface antigen (HBsAg) serves as an indicator of the transcriptional activity of cccDNA [5, 6]. Achieving a “functional cure,” marked by HBsAg removal along with a persistent virological response, loss of hepatitis B e-antigen (HBeAg), recovery of alanine aminotransferase (ALT), and improved liver tissue lesions, is considered the optimal treatment goal [1, 6].

The current drug management for HBsAg loss falls into two main categories: nucleoside analogues (NAs) and pegylated interferon alpha (PegIFN) [3, 7]. While NAs provide cost-effective benefits, they lacked direct targeting of cccDNA, necessitating extended or indefinite treatment courses with potential issues of drug resistance [8]. Conversely, PegIFN can significantly decrease HBsAg levels in specific patient groups [9], potentially enhancing the HBsAg elimination rate when combined with or transitioned from NAs [10, 11]. However, the HBsAg clearance rate varies when PegIFN is combined with NAs, influenced by different NA subtypes and individual genes [12, 13].

To expedite the early discovery of effective treatments, it is crucial to identify additional response markers measurable during ongoing treatment. Thus, our study aims to evaluate the predictive ability of serum indicators for HBsAg clearance IN CHB patients treated with NAs and PegIFN during 48-week follow-up.

## Methods

### Patients

**Fig. 1:**
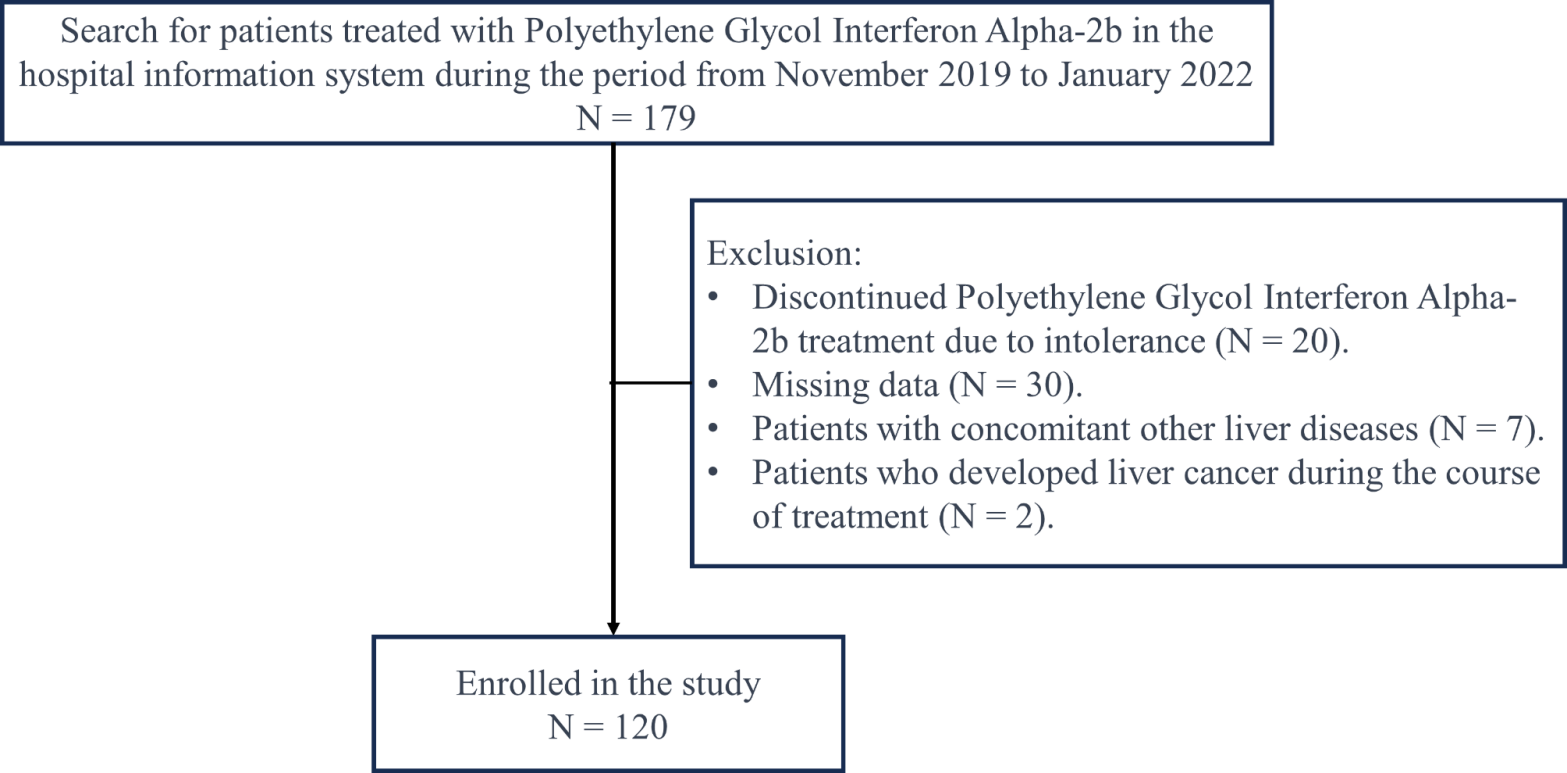
The study flowchart

We studied 179 consecutive patients treated with NAs and PegIFN-2b at our institution from November 2019 to January 2022. The inclusion criteria were as follows: [1] individuals must be 18 years of age or older; [2] they must have tested positive for HBsAg, HBV DNA, or both for a period exceeding six months. The exclusion criteria encompassed: [1] the concurrent presence of other forms of viral hepatitis; [2] the coexistence of other liver conditions such as autoimmune liver disease or liver injury induced by drugs; [3] instances where clinical data collection was incomplete. A total of 20 participants withdrew from the study due to drug intolerance, with none experiencing HBsAg seroreversion. After excluding 59 patients not meeting criteria, 120 eligible patients remained (Fig. 1). This study has been authorized by the ethics committee of the Third Affiliated Hospital of Wenzhou Medical University (YJ2024052). Written informed permission was obtained from all individuals.

### Data collection

Clinical information and laboratory tests, including ALT, aspartate aminotransferase (AST), white blood cells (WBC), PLT, absolute neutrophil value, and HBsAg, were collected at baseline, 12- and 24-weeks during treatment, with additional HBsAg data at 48 weeks.

### Evaluation of the liver with Ultrasound

Before therapy, patients underwent liver ultrasound at the ultrasound imaging center following an 8-hour fasting period. The scoring system, based on echo and contour, assigned 0–2 points to the liver ultrasound [14].

### Endpoint

HBsAg levels < 0.05 IU/mL at 48 weeks indicated clearance, signifying clinical cure (CC group). HBsAg levels ≥ 0.05 IU/mL indicated no-clinical cure (No-CC group). We have established a set of pre-specified criteria to guide us in deciding whether to discontinue antiviral treatment in the event of adverse drug reactions. These criteria are formulated based on clinical practice guidelines and data from drug safety monitoring, ensuring that we can respond promptly and appropriately to any potential adverse reactions [15].

### Statistical analysis

Categorical variables were expressed as percentages. Group comparisons used the chi-square test. Mean ± standard deviation described normal distribution data, analyzed with the t-test, while skewed distribution data used the median (interquartile range) with non-parametric testing (such as HBsAg). Logistic regression identified independent factors influencing clinical cure. For the logistic regression analysis, variable selection was guided by clinical significance, theoretical considerations, and empirical evidence from prior research. We began with a univariate screening to identify potential predictors associated with the outcome at a p-value threshold of 0.25. Variables were then entered into a stepwise multivariate model, retaining those with *p* < 0.05 for the final model. Multicollinearity was assessed using the variance inflation factor (VIF), and variables with high correlation were considered for exclusion to ensure model stability. The model’s goodness-of-fit was evaluated using the Hosmer-Lemeshow test, and diagnostics were performed to check for linearity, independence of errors, and the presence of influential outliers. The predictive accuracy of the formula was assessed using the ROC curve at different time points (before therapy, 12 weeks, and 24 weeks into treatment). A P-value below 0.05 was considered statistically significant, with SPSS 20.0 used for analysis.

## Results

### Baseline characteristics of enrolled patients

A comprehensive review was conducted on 179 individuals who underwent PegIFN-2b treatment at our hospital from November 2019 to January 2022, identified through a search of the hospital information system. After excluding 59 individuals who did not meet the criteria, the analysis was based on 120 eligible patients who received NAs therapy and completed a 48-week course of PegIFN-2b (Fig. 1). Among the cohort of patients under study, 71 (59.1%) patients had CC while 49 (40.9%) patients did not (Table 1). There were 88 male and 32 female individuals, aged between 22 and 46 years, with an average age of 36.98 ± 7.540 years. The median level of HBsAg was 621.6 IU/ml, ranging from 258.0 to 1658.0 IU/ml in No-CC group, while 25.8 (4.0, 189.6) IU/ml in CC group. The ALT level and AST lever did not differ between two groups (*P* > 0.05). The more detailed information was showed in Table 1.

**Table 1:** Baseline characteristics of the patients

Variables	No-CC group	CC group	*P* value
	*N* = 49	*N* = 71	
Sex, N (%)			0.856
Male	35 (71.4)	53 (74.6)	
Female	14 (28.6)	18 (25.4)	
Age, years old	40. 4 (8. 5)	34.7 (5.8)	< 0.001
Alanine aminotransferase, U/L (Normal range 9–50 U/L)	31.0 (27.0, 37.0)	37.7 (23.5, 61.0)	0.068
Aspartate aminotransferase, U/L (Normal range 15–40 U/L)	31.0 (26.0, 37.0)	30.0 (22.5, 45.4)	0.685
HBsAg, IU/ml	621.6 (258.0, 1658.0)	25.8 (4.0, 189.6)	< 0.001
HBeAg, IU/ml	0.5 (0.4, 0.8)	0.38 (0.3, 0.5)	0.02
HBeAb, IU/ml	0.005 (0.004, 0.006)	0.004 (0.003–0.005)	0.070
White blood cell, 10^9^/L	5.4 (1. 6)	5.2 (1.6)	0.414
Platelet, 10^12^/L	196.5 (59.2)	179.5 (64.2)	0.145
Neutrophils, 10^9^/L	2.8 (1.1)	2.7 (1.1)	0.401
Lymphocyte, 10^9^/L	2.1 (0.7)	1.9 (0.5)	0.035
AFP (Normal range 0–7.0 ng/ml)	5.6 (11.9)	3.1 (2.5)	0.092
Liver ultrasound scores, N (%)			0.035
0	42 (85.7)	47 (66.2)	
1	6 (12.2)	15 (21.1)	
2	1 (2.0)	9 (12.7)	

Abbreviation: CC = clinical cure; HBsAg = Hepatitis B surface antigen

Categorical variables were expressed as percentages. Group comparisons used the chi-square test. Mean ± standard deviation described normal distribution data, analyzed with the t-test, while skewed distribution data used the median (interquartile range) with non-parametric testing

### Dynamic changes in serological indicators

In the analysis of outcomes following 48 weeks of pegylated interferon therapy for patients with hepatitis B, 71 cases demonstrated CC, while 49 cases did not achieve such outcomes. At 12 weeks, only serum HBsAg between CC group and No-CC group had statistical difference, while at 24 weeks, all presented indications were not (Table 2). Furthermore, a noteworthy distinction was identified in the proportion of patients achieving a 1-log reduction in HBsAg at 24 weeks (Table 2).

**Table 2:** Dynamic changes in serological indicators

Features	At 12 weeks			At 24 weeks		
	CC-group	No-CC group	P value	CC-group	No-CC group	*P* value
	(*N* = 71)	(*N* = 49)		(*N* = 71)	(*N* = 49)	
White blood cell, 10^9^/L	3.2(2.5,3.6)	3.2(2.7,3.8)	0.326	3.3(2.59,3.81)	3.22(2.5,3.6)	0.232
Platelet, 10^12^/L	108(87,126)	119(93,134.5)	0.233	113(88,136)	112(89,144)	0.913
Neutrophils, 10^9^/L	1.49(1.19,1.8)	1.4(1.1,1.78)	0.513	1.5(1.0,2.0)	1.3(1.1,1.7)	0.394
Aspartate aminotransferase, U/L	57(45,80)	34.5(27.9,42.5)	0.0275	30(25,42)	31(25.4,39.5)	0.533
Alanine aminotransferase, U/L	128(68,162)	36(26,50)	< 0.001	45(33.6,75)	39.1(26,60)	0.774
HBsAg decreased by > 1 log(IU/ml) compared to baseline, N (%)	9(12.68)	2(4.08)	0.200	40(56.34)	16(32.65)	0.011

Abbreviation: CC = clinical cure; HBsAg = Hepatitis B surface antigen

Categorical variables were expressed as percentages. Group comparisons used the chi-square test. Mean ± standard deviation described normal distribution data, analyzed with the t-test, while skewed distribution data used the median (interquartile range) with non-parametric testing

### Prediction model

Through multivariate analysis, statistically significant differences were observed between the groups achieving clinical cure and those without in parameters including age, pre-treatment quantitative HBsAg levels, as well as ALT and AST levels at 12 weeks, and pre-treatment abdominal ultrasound scores (Table 3). Nevertheless, factors such as gender, WBC, absolute neutrophil value, platelet count at treatment initiation, and abdominal ultrasound scores at 12 and 24 weeks did not exhibit statistically significant differences between the groups achieving clinical cure and those without (*P* > 0.05).

**Table 3:** Variables in prediction models

Variables	At baseline		12 weeks		24 weeks	
	OR (95% CI)	*P* value	OR (95% CI)	*P* value	OR (95% CI)	*P* value
Age	0.820 (0.747–0.901)	0.001	0.879 (0.816–0.947)	0.001	0.885 (0.831–0.943)	0.001
HBsAg at baseline	0.998 (0.997–0.999)	0.001	4.380 (1.237–15.508)	0.022	2.977 (1.300-6.818)	0.001
Liver ultrasound scores at baseline	6.600 (1.964–22.180)	0.002	3.227 (0.902–11.544)	0.072	-	-

Abbreviation: HBsAg = Hepatitis B surface antigen

We incorporated differential factors at pre-treatment, 12 weeks, and 24 weeks into Logistic regression analysis. At the onset of treatment, age, HBsAg, and abdominal ultrasound score emerged as independent predictors for clinical cure of hepatitis B. Subsequently, using the identified independent predictors, we constructed a pre-treatment predictive model = 8.592 + 1.887 × Abdominal ultrasound score − 0.002 × HBsAg − 0.198 × Age. At 12 weeks of treatment, ALT and age were identified as independent influencing factors for clinical cure in hepatitis B patients (Table 3). Based on these independent factors, we developed a predictive model = 3.763 + 1.477 × 12-week ALT − 0.129 × Age. Moving to the 24-week mark, age and whether HBsAg decreased > 1 log from baseline were identified as independent influencing factors for clinical cure at 48 weeks in hepatitis B (Table 3). Consequently, we utilized the selected independent factors to construct a predictive model = 4.443 + 1.091 × (HBsAg decrease > 1 log from baseline) − 0.122 × Age.

**Fig. 2:**
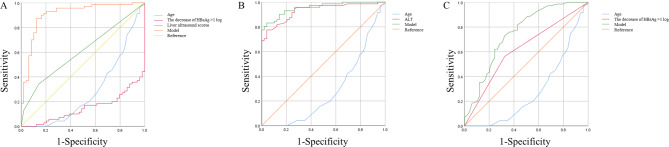
The ROC curves assessing the probability of clinical cure at 48 weeks for chronic hepatitis B patients undergoing interferon therapy at different time points

The ROC curves assessing the probability of clinical cure at 48 weeks for CHB patients undergoing interferon therapy at different time points were depicted in Fig. 2. For the pre-treatment predictive model, ROC analysis yielded an AUC value of 0.907, with a standard error of 0.030 and a 95% confidence interval (CI) of 0.848–0.966. The optimal cutoff value was 0.534, yielding a Youden index of 0.749, sensitivity of 87.1%, and specificity of 87.8% (Fig. 2A). The 12-week predictive model, through ROC analysis, yielded an AUC value of 0.958, with a standard error of 0.016 and a 95% CI of 0.927–0.989. The optimal cutoff value was 0.414, with a Youden index of 0.752, sensitivity of 91.5%, and specificity of 83.7% (Fig. 2B). Lastly, the 24-week predictive model, through ROC analysis, yielded an AUC value of 0.747, with a standard error of 0.047 and a 95% CI of 0.654–0.839. The optimal cutoff value was 0.558, with a Youden index of 0.405, sensitivity of 73.2%, and specificity of 67.3% (Fig. 2C).

## Discussion

The current study’s findings indicated that the combination of PegIFNα and NAs demonstrated efficacy in treating patients with CHB, aligning with the predictive formulas for clinical cure that we developed and providing valuable insights into potential treatment strategies. However, the study design does not include comparisons with other treatment modalities, and thus, it is premature to conclude the efficacy of this combination therapy over monotherapy with NAs. Further research is necessary to validate the effectiveness of this combined approach and to compare it with the efficacy of monotherapy with NAs.

Numerous research studies have underscored the impact of age and gender on the effectiveness of interferon, particularly in the context of treating HBV. Notably, younger individuals and females have exhibited more favorable outcomes, demonstrating higher rates of HBsAg negative conversion. In the specific investigation conducted herein, individuals within the HBV cured group were observed to be notably younger than their uncured counterparts, a distinction that was statistically significant. Further analysis through multivariate regression, both before treatment initiation and at 12 and 24 weeks into the therapeutic regimen, consistently identified age as a statistically significant variable. This underscores its role as an independent influencing factor in predicting the clinical cure of CHB patients. The data strongly suggest that younger patients enjoy a distinct advantage over their older counterparts in achieving favorable treatment outcomes. An additional examination of interferon α treatment for CHB within a domestic context found that this therapeutic modality yielded more positive results among young women [16]. Nevertheless, the current study did not reveal a significant disparity between men and women in terms of treatment outcomes.

In the NEW-SWITCH study [17], a detailed subgroup analysis was undertaken to investigate the impact of baseline serum HBsAg levels on the clearance rates during treatment durations of 48 and 96 weeks. Notably, patients with baseline serum HBsAg < 1500 IU/ml exhibited a substantially higher clearance rate compared to those with baseline serum HBsAg ≥ 1500 IU/ml, irrespective of treatment duration (48-week group: 26.5% vs. 4.7%, *P* < 0.05; 96-week group: 40.0% vs. 3.8%, *P* < 0.05). This discerned disparity underscores the prognostic value of lower baseline serum HBsAg levels in predicting favorable HBsAg clearance outcomes. Concerning factors influencing serum HBsAg clearance, extant literature has consistently identified the decline in HBsAg levels during treatment as a predictive indicator. Studies on inactive HBsAg carriers (IHC) have demonstrated that an HBsAg reduction of > 0.6 log from baseline at 24 weeks correlates with an impressive 84% clearance rate [18]. Brunetto MR et al. posit that a reduction in HBsAg levels exceeding 1 log IU/mL during treatment significantly associates with subsequent HBsAg clearance [19]. Consequently, these findings accentuate the clinical relevance of assessing HBsAg levels after 24 weeks of treatment in prognosticating treatment outcomes and discerning patients with heightened prospects of clinical cure.

ALT is localized within the cytoplasm of hepatocytes and stands as a highly sensitive indicator for detecting liver damage. Its release into the bloodstream is triggered by either liver cell damage or an increase in the permeability of the liver cell membrane. Consequently, heightened levels of ALT in the blood serve as a reflective marker of liver injury. Likewise, AST is widely employed in liver function testing, serving as an additional indicator of liver function impairment. Elevated levels of ALT and AST concurrently signify the extent of inflammation within liver cells, providing insights into the body’s response to hepatic stressors. A domestic investigation exploring factors influencing clinical outcomes in 107 HBeAg-negative patients [19] revealed a noteworthy surge in ALT levels during the fourth week of interferon treatment. This elevation emerged as a predictive factor for subsequent HBsAg clearance post-treatment. Consistent with broader research on interferon therapy, multiple studies have established a correlation between increased serum ALT and AST levels and the treatment response of patients [20–22]. In the current study, single-factor analysis during the 12-week course of interferon treatment unveiled a positive association between the elevation of patients’ ALT and AST levels and an augmented clinical cure rate for hepatitis B, a relationship supported by statistical significance (*P* < 0.05). The underlying rationale for this phenomenon is posited to be intricately linked to the host’s immune response triggered by interferon administration. Subsequent logistic regression analysis substantiated the role of ALT elevation at the 12-week mark as an independent influencing factor in predicting clinical cure among patients with CHB. The analysis revealed a robust association, with an odds ratio of 4.380 (95% confidence interval: 1.237–15.508, *P* = 0.022), signifying the capacity of ALT elevation at this juncture to prognosticate the likelihood of achieving clinical cure within the 48-week treatment period. This nuanced insight underscores the clinical relevance of monitoring ALT levels as a predictive biomarker in the trajectory of interferon-based therapeutic interventions for CHB.

Nevertheless, it is imperative to underscore that the utility of these prediction models necessitates further comprehensive validation to ascertain their generalizability across diverse populations and various medical institutions. Furthermore, an enhanced exploration of the interpretability of these models is warranted to facilitate a more profound understanding of their clinical implications, thereby empowering healthcare professionals and patients alike. The ongoing refinement and validation of these models hold the potential to usher in a new era of personalized management for CHB treatment, promising positive implications for their integration into routine clinical practice. Diverse patients exhibit varying tolerances to the adverse reactions associated with interferon treatment. Vigilant monitoring and close follow-up on the effects and potential adverse reactions of related treatments during interferon therapy are paramount [23]. However, this study has several limitations. Firstly, the observational study design was inherently limited by factors such as selection bias, information bias, and the difficulty in controlling for all potential confounding factors related to the outcome. These factors could lead to incorrect inferences about causality. Secondly, some patients discontinued therapy due to toxicity, which might introduce further bias. Additionally, our research did not include patients who underwent liver biopsies. We suggested that future research incorporating transient elastography and liver biopsies would be valuable for validating and expanding upon our findings.

## Conclusion

In conclusion, our investigation introduces novel and reliable prediction formulas, shedding light on factors influencing clinical cure at various treatment stages. These findings hold practical implications for predicting clinical cure rates among CHB patients treated with nucleoside analogs, while also endorsing the efficacy and safety of PegIFNα-2b. Recognizing the limitations of our single-center retrospective study, we advocate for future endeavors comprising multi-center, prospective, large-sample cohort studies to further elucidate the complex factors contributing to HBV cure.

## Data Availability

The datasets used and/or analyzed during the current study available from the corresponding author on reasonable request.

## Abbreviations

CHB: Chronic hepatitis B
NAs: Nucleoside analogues
PegIFN: Pegylated interferon alpha
ROC: Receiver operating characteristic
HBsAg: Hepatitis B surface antigen
cccDNA: Covalently closed circular DNA
ALT: Alanine aminotransferase
CC: Clinical cure
